# Draft Genome Sequence of Vibrio owensii GRA50-12, Isolated from Green Algae in the Intertidal Zone of Eastern Taiwan

**DOI:** 10.1128/genomeA.01438-14

**Published:** 2015-01-15

**Authors:** Ling-Chun Lin, Guang-Huey Lin, Yi-Hsiung Tseng, Mei-Shiuan Yu

**Affiliations:** a Department of Microbiology, School of Medicine, Tzu Chi University, Hualien, Taiwan; b Institute of Medical Sciences, Tzu Chi University, Hualien, Taiwan

## Abstract

Vibrio owensii GRA50-12 was isolated from symbiotic green algae of coral. The genome contains genes encoding toxin production, virulence regulation, stress response proteins, types II, IV, and VI secretion systems, and proteins for the metabolism of aromatic compounds, which reflects its pathogenic potential and its ecological roles in the ocean.

## GENOME ANNOUNCEMENT

Vibrio owensii is a Gram-negative marine bacterium belonging to the Harveyi clade. This organism is a potential pathogen, which kills crustacean larvae (1, 2) and causes a coral disease, Montipora white syndrome (3). Symbiotic green algae were collected from coral from an intertidal zone in eastern Taiwan. An algal homogenate was prepared, and V. owensii GRA50-12 in the homogenate was isolated by plating bacteria in the homogenate on thiosulfate-citrate-bile salts-sucrose agar. We report here a draft genome sequence of V. owensii GRA50-12.

The V. owensii GRA50-12 genomic DNA was extracted using a WelPrep DNA kit (catalog no. D001; Welgene Biotech) according to the manufacturer’s instructions. DNA was sequenced by the Illumina Solexa pyrosequencing system and underwent a filtering process to obtain the qualified reads (4). ConDeTri was used to trim or remove the reads according to the quality score. Nuclear reads were assembled *de novo* using ABySS (5), which yielded around 700-fold coverage. The number of generated contigs is 51. Genome annotations were created in MAKER 2.00 (6) using a GeneMark (7) model trained for V. owensii GRA50-12 via self-training. The resulting predictions were searched against the NCBI nonredundant (nr) database by using BLASTp. A general analysis of the coding genes, their functional classification, and pathway reconstruction was performed by Rapid Annotations using Subsystems Technology (RAST) (8) and a KAAS sever (9). The genome consists of 6,006,497 bp, with a G+C content of 45%. The number of coding sequences is 4,891. Noncoding RNA genes were identified by Aragorn (10) and RNAmmer (11), which identified 120 tRNAs, 1 transfer-messenger RNA (tmRNA), and 22 rRNAs.

Based on the analysis performed using multilocus sequence analysis (MLSA), Karlin genomic signature, and average amino acid identity (ANI) (http://enve-omics.ce.gatech.edu/ani/), we determined the taxonomic position of the V. owensii GRA50-12 strain according to the genome-based taxonomy for vibrios (12), which revealed >98% DNA identity in MLSA, 1 in Karlin signature, and >97% ANI with V. owensii strains 25919 (accession no. BANZ00000000), CAIM 1854 (accession no. BAOH00000000), and LMG 25430 (accession no. BAOE00000000).

Functional assignment and metabolic pathway mapping predicted that this draft genome sequence contains 118 genes involved in virulence and defense. These genes include those encoding hemolysin, colicin V, and bacteriocin production. The membrane transport apparatus includes type II, IV, and VI secretion systems, and a number of ABC transporters were also identified, although the genome lacks a type III secretion system that is commonly present in vibrios. The genes associated with virulence regulation and cell signaling are present in high numbers. A *barA-sirA* two-component system, which promotes the expression of virulence genes and decreases the expression of motility genes (13), as well as a three-component system that is required for activating the cholera toxin-encoding operon, *ctxAB*, were predicted (14). The strain also contains genes coding for the catabolism of aromatic compounds and for stress responses, such as osmotic shock and oxidative stress. These genes confer a niche fitness and virulence regulation for infection and also benefit the survival of this marine bacterium in the open ocean and in interaction with its symbiotic hosts.

### Nucleotide sequence accession numbers.

This whole-genome shotgun project has been deposited in DDBJ/EMBL/GenBank under accession numbers BBPJ01000001 to BBPJ01000051. The version described in this paper is the first version.
